# ﻿Three new species of *Isodon* (Nepetoideae, Lamiaceae) from China

**DOI:** 10.3897/phytokeys.246.130432

**Published:** 2024-09-19

**Authors:** Ya-Ping Chen, Hua Peng, Alan J. Paton, Chun-Lei Xiang

**Affiliations:** 1 CAS Key Laboratory for Plant Diversity and Biogeography of East Asia, Kunming Institute of Botany, Chinese Academy of Sciences, Kunming 650201, China Kunming Institute of Botany, Chinese Academy of Sciences Kunming China; 2 Royal Botanic Gardens, Kew, Richmond TW9 3AE, UK The Royal Botanic Gardens Richmond United Kingdom; 3 Key Laboratory of Phytochemistry and Natural Medicines, Kunming Institute of Botany, Chinese Academy of Sciences, Kunming 650201, China Kunming Institute of Botany Kunming China

**Keywords:** dry valley, Hengduan Mountains, Isodoninae, Ocimeae, southwest China

## Abstract

Three new species of *Isodon* (Lamiaceae) from China are described and illustrated, based on both morphological evidence and our recent phylogenomic studies of the genus. *Isodonattenuatus*, a herbaceous new species known only from the Fanjing Mountain, is shown to be sister to *I.villosus*, but they can be easily distinguished by leaf and inflorescence indumentum, calyx teeth shape and corolla tube morphology. *Isodongongshanensis*, a herbaceous new species collected from the Hengduan Mountains in southwest China, represents a distinct lineage within the genus. *Isodonsukungii*, a shrubby new species also endemic to the Hengduan Mountains, was previously misidentified as *I.tenuifolius*, but they are phylogenetically distantly related and differ in lamina size and margin, inflorescence type and corolla length and shape.

## ﻿Introduction

*Isodon* (Schrad. ex Benth.) Spach (Ocimeae, Nepetoideae, Lamiaceae) is a genus of approximately 140 species mainly distributed in subtropical to tropical Asia, with two disjunct species endemic to Africa (Wu and Li 1977; Li 1988; Li and Hedge 1994). The genus is most diverse in southwest China, particularly in the dry valleys in the Hengduan Mountains (Zhong et al. 2010; Yu et al. 2014; Chen et al. 2022). Morphologically, *Isodon* can be distinguished from other genera of tribe Ocimeae by its pedunculate and bracteolate cymes, slightly or strongly 2-lipped (3/2) calyces, strongly 2-lipped (4/1) corollas and free filaments inserted at the base of the corolla tube (Li 1988; Paton and Ryding 1998; Harley et al. 2004).

Resolving the intrageneric relationships within *Isodon*, based on limited DNA loci (Zhong et al. 2010; Yu et al. 2014; Chen et al. 2019) or plastome sequences (Chen et al. 2022) has been difficult due to the rapid radiation of the genus in southwest China. Recently, we reconstructed a robust phylogeny for 126 *Isodon* taxa using transcriptome and genome-resequencing data (Chen et al. 2024). Except for the four clades (Clade I–Clade IV) recovered consistently in previous molecular phylogenetic studies (Yu et al. 2014; Chen et al. 2019, 2022), four subclades (Clade IVa–Clade IVd) were further recognised within the largest Clade IV which comprises ca. 80% species of the genus (Chen et al. 2024). Meanwhile, Chen et al. (2024) confirmed the statuses of three unidentified species we collected during 2018–2020 as new to science. These species were thus named *I.attenuatus* Y.P.Chen & C.L.Xiang, *I.gongshanensis* Y.P.Chen & C.L.Xiang and *I.sukungii* Y.P.Chen & C.L.Xiang and described below.

## ﻿Materials and methods

Phylogenetic placements of the three new species within *Isodon* were directly referenced from our recent phylogenomic study of the genus (Chen et al. 2024). Morphological comparisons of these new species and other *Isodon* taxa were conducted, based on our previous field observations, specimen examination and morphological investigations of mericarps (Chen et al. 2022). Specimens from 17 Herbaria (BM, CDBI, E, GXMI, HIB, IBK, IBSC, K, KUN, KYO, LE, MW, NAS, PE, SZ, TI and WUK; abbreviations follow Thiers 2024) and our field collections were examined. Additionally, images of specimens (including type specimens) and living plants of *Isodon* from JSTOR (https://www.jstor.org/), Global Biodiversity Information Facility (GBIF, https://www.gbif.org/), Chinese Virtual Herbarium (CVH, https://www.cvh.ac.cn/) and Plant Photo Bank of China (PPBC, http://ppbc.iplant.cn/) were checked. Furthermore, protologues of all published names and related taxonomic and floristic literature on *Isodon* were reviewed. Morphological descriptions of the new species followed the terminology used by Li (1988) and Li and Hedge (1994).

## ﻿Results and discussion

**Figure 1.**
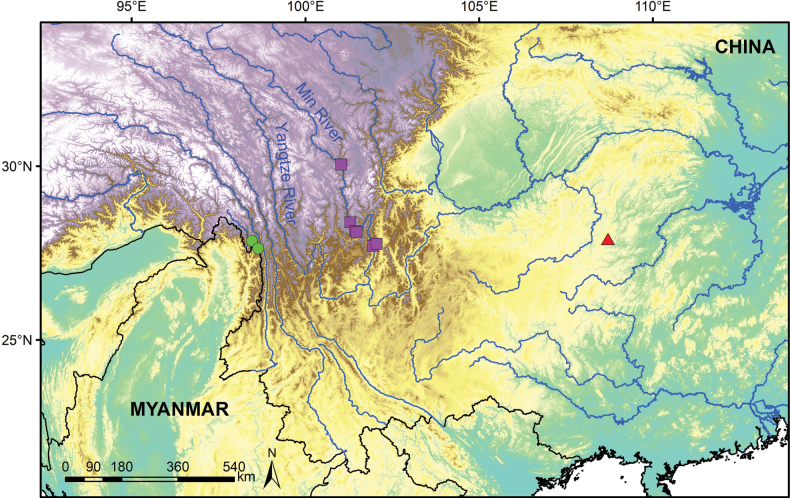
Geographic distribution of *Isodonattenuatus* (red triangle), *I.gongshanensis* (green circles) and *I.sukungii* (purple squares).

**Figure 2.**
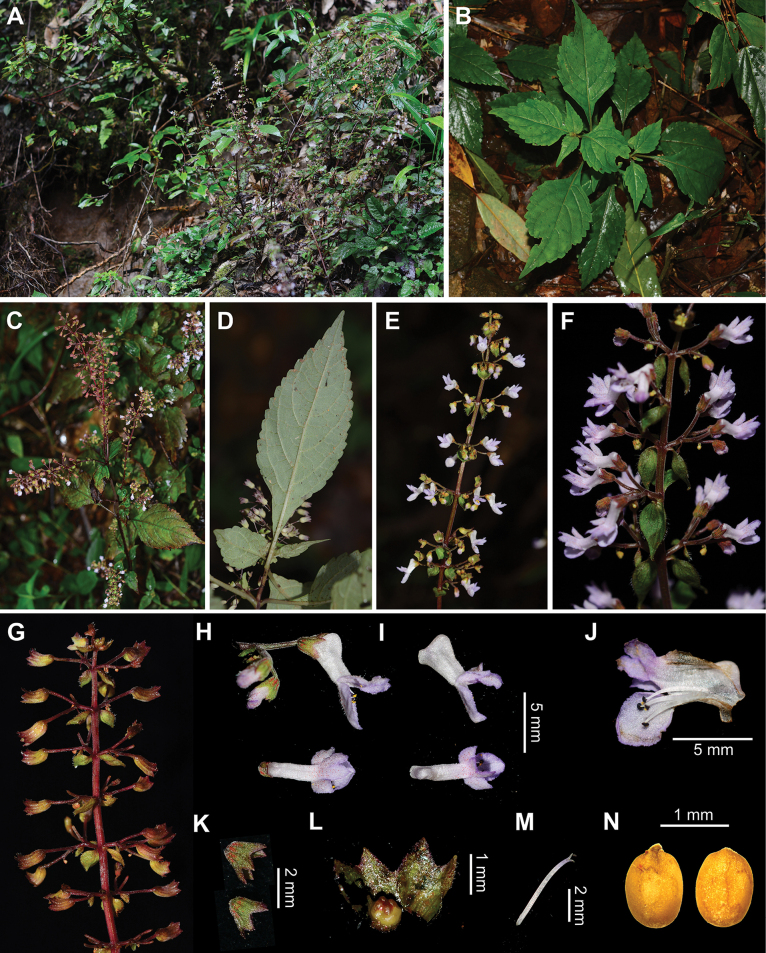
Morphology of *Isodonattenuatus* from the type locality **A** habitat **B, C** habit **D** abaxial view of lamina **E, F** inflorescences **G** infructescence **H** flowers **I** corollas **J** dissected corolla **K** calyces **L** ovary **M** style **N** mericarps (Photographed by Ya-Ping Chen).

*Isodonattenuatus* (corresponding to *Isodon* sp. 1 in Chen et al. (2024)) was collected from the Fanjing Mountain in Guizhou Province, China (Fig. 1) and was recovered in Clade I according to Chen et al. (2024). It shares reddish-brown glands covering the plants as in other taxa of Clade I, a characteristic considered a synapomorphy of this clade (Zhong et al. 2010; Yu et al. 2014; Chen et al. 2022). Phylogenetically, *I.attenuatus* is sister to *I.villosus* Y.P.Chen & H.Peng (Chen et al. 2024) from the adjacent Guangxi Zhuang Autonomous Region. Both species have a declinate corolla, lobes of posterior corolla lip with an acute apex and stamens and style included within the corolla (Fig. 2), differing from the straight corolla, rounded apices of the lobes of posterior corolla lip and/or exserted stamens and style of other taxa of Clade I. However, they can be easily differentiated by the morphology of lamina, which is subglabrous with a decurrent base in *I.attenuatus* (Fig. 2), but densely villose with a non-decurrent base in *I.villosus*. There are also differences in the calyx teeth shape, with *I.attenuatus* having triangular teeth with an acute apex and *I.villosus* having ovate teeth with an obtuse apex. Furthermore, the corolla tube of *I.attenuatus* is significantly attenuate towards the throat, but that of *I.villosus* is not attenuate. More detailed differences between the two species are listed in Table 1. *Isodonattenuatus* is now only known from its type locality, where it grows along hiking trails in subtropical evergreen broadleaf forests. It is possibly threatened by human disturbances, such as tourism and herbicide use. However, more comprehensive investigations are needed to further elucidate its distribution, decline and conservation status.

**Table 1.** Morphological comparisons between *Isodonattenuatus* and *I.villosus*.

Characters	*I.attenuatus*	*I.villosus*
Lamina	Subglabrous, base cuneate to broadly cuneate, decurrent	Densely to sparsely villose, base broadly cuneate to shallowly cordate, not decurrent
Inflorescence	Densely puberulent and glandular puberulent	Densely villose and glandular puberulent
Pedicel	4–6 mm long	2.5–4 mm long
Fruiting calyx	Teeth triangular, apex acute, not folded	Teeth ovate, apex obtuse, folded
Corolla	Tube attenuate towards the throat, lips light bluish-purple	Tube not attenuate, lips white

**Figure 3.**
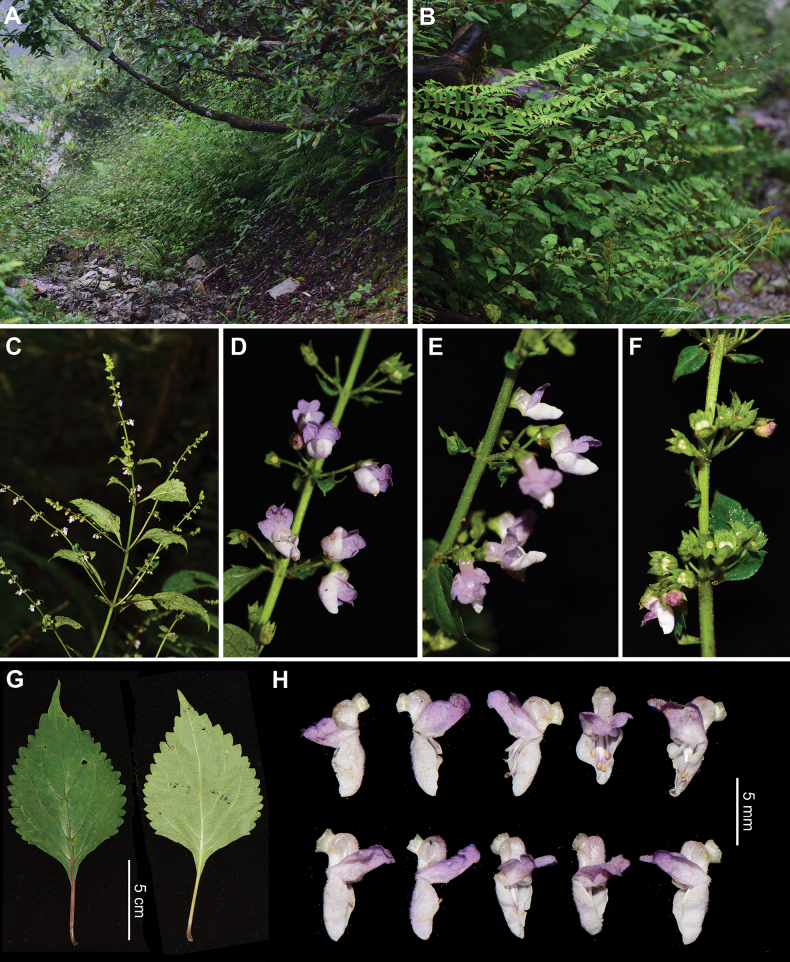
Morphology of *Isodongongshanensis* from the type locality **A** habitat **B** habit **C–E** inflorescences **F** infructescence **G** leaves **H** corollas (Photographed by Ya-Ping Chen).

*Isodongongshanensis* is endemic to Gongshan County in Yunnan Province, southwest China (Fig. 1). It typically grows in mixed needle-leaf and broadleaf forests in the Gaoligong Mountain at altitudes of 2700–2900 m. The new species (corresponding to *Isodon* sp. 11 in Chen et al. (2024)) was identified as a distinct lineage within Clade IVc, serving as the sister group to the remaining herbaceous taxa in this clade (Chen et al. 2024). Morphologically, *I.gongshanensis* is most similar to *I.rosthornii* (Diels) Kudô from the sister clade. While they share similar indumentum and inflorescence types, they exhibit clear differences in the morphology of the calyx and corolla. The calyx teeth of the posterior lip of *I.gongshanensis* are lanceolate and ca. 1 mm long (Fig. 3), in contrast to the triangular teeth of *I.rosthornii*, which are about 0.5 mm long. In *I.gongshanensis*, the corolla is 6–7 mm long with a pink posterior lip lacking spots, whereas in *I.rosthornii*, the corolla is ca. 5 mm long with a white posterior lip marked by reddish-purple spots. Additionally, the ovary of *I.gongshanensis* is glandless, whereas that of *I.rosthornii* is glandular. Other differences between the two species are summarised in Table 2.

**Table 2.** Morphological comparisons between *Isodongongshanensis* and *I.rosthornii*.

Characters	*I.gongshanensis*	*I.rosthornii*
Lamina	Ovate to broadly ovate, apex acuminate, base cuneate to broadly cuneate	Broadly ovate to subrounded, apex acute to obtuse, base broadly cuneate to subrounded
Calyx	2-lipped to 1/2 its length, teeth of the posterior lip ca. 1 mm long, narrowly triangular, apex acuminate	2-lipped to over 1/2 its length, teeth of the posterior lip ca. 0.5 mm long, triangular, apex acute
Corolla	6–7 mm long, posterior lip pink without spots	Approximately 5 mm long, posterior lip white with reddish-purple spots
Ovary	Non-glandular	Glandular

**Figure 4.**
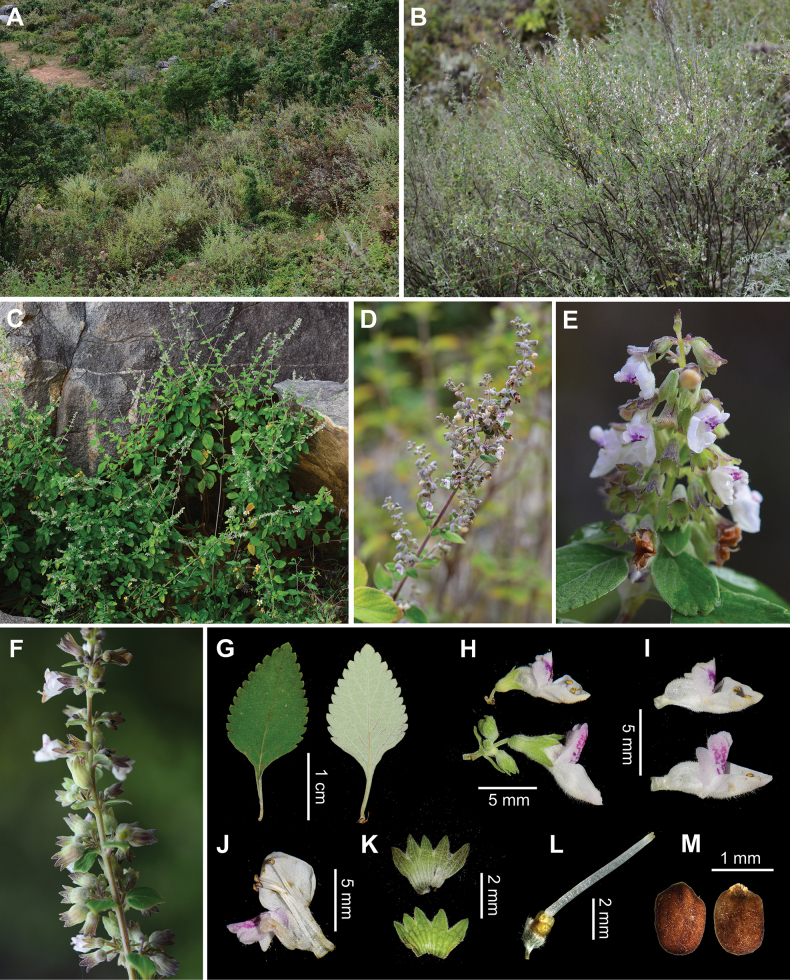
Morphology of *Isodonsukungii* from the type locality **A** habitat **B, C** habit **D, E** inflorescences **F** infructescence **G** leaves **H** flowers **I** corollas **J** dissected corolla **K** dissected calyces **L** pistil **M** mericarps (Photographed by Ya-Ping Chen).

*Isodonsukungii* is a shrubby species restricted to the dry valley along Min River in the Hengduan Mountains (Fig. 1). It was previously misidentified as *I.tenuifolius* (W.W.Sm.) Kudô from a geographically adjacent, but isolated valley along the upper Yangtze River, perhaps due to the small and densely grey tomentose leaves shared by both species (Fig. 4). However, phylogenetic analysis revealed that the two species are distantly related, despite both being placed in Clade IVd (Chen et al. 2024). *Isodonsukungii* (corresponding to *Isodon* sp. 5 in Chen et al. (2024)) is phylogenetically most closely related to morphologically distinct species from the same river valley. The small and densely grey tomentose leaves of *I.sukungii and I.tenuifolius* may represent a case of convergent evolution and adaptation to dry habitats. The two species can be distinguished by their lamina length and margin: in *I.sukungii*, the lamina is 1–4 cm long and serrate, whereas in *I.tenuifolius*, it is 0.5–1 cm long and usually entire. The cymes in *I.sukungii* often form panicles, while in *I.tenuifolius*, they are simple and never arrange into a thyrse or panicle. Additionally, the corolla of *I.sukungii* is 6–7 mm long with a straight anterior lip, compared to the ca. 4 mm long corolla with a strongly reflexed anterior lip in *I.tenuifolius*. Other minute differences between the two species are detailed in Table 3.

**Table 3.** Morphological comparisons between *Isodonsukungii* and *I.tenuifolius*.

Characters	*I.sukungii*	*I.tenuifolius*
Lamina	Ovate to oblong, thick papery, 1–4 × 0.5–2 cm, margin serrate	Oblong to subrounded, papery, 0.5–1 × 0.4–0.7 cm, margin entire, rarely serrate
Petiole	0.3–1 cm long	1–3 (–5) mm long
Cymes	Forming secund panicles up to 20 cm long	Single, not forming thyrses or panicles
Corolla	6–7 mm long, tube exerted from the calyx, anterior lip straight	Approximately 4 mm long, tube included within the calyx, anterior lip strongly reflexed
Mericarp	Surface glabrous	Surface sparsely glandular

### ﻿Taxonomic treatment

#### ﻿ Isodon attenuatus

Taxon classificationPlantaeLamialesLamiaceae

Y.P.Chen & C.L.Xiang sp. nov.

F446AB18-5045-50BA-82F9-3C20787F72CF

urn:lsid:ipni.org:names:77348680-1

Fig. 2

##### Type.

China – **Guizhou Prov.** • Jiangkou County, Fanjing Mountain, along the hiking trail in forests; 27°53'45.08″N, 108°42'39.09″E; alt. 1635 m; 1 Sep 2018; *Y.P. Chen et al. EM590* (holotype: KUN1628213!; isotypes: K!, KUN1628215!, PE!).

##### Diagnosis.

*Isodonattenuatus* is most closely related to *I.villosus*, but differs by having subglabrous (vs. densely to sparsely villose) laminae with a decurrent (vs. not decurrent) base, densely puberulent and glandular puberulent (vs. densely villose and glandular puberulent) inflorescences, longer pedicels (4–6 mm vs. 2.5–4 mm long), triangular (vs. ovate) calyx teeth and a corolla tube attenuate towards the throat (vs. not attenuate).

Herbs perennial, 30–70 cm tall. Rhizomes woody, tuberose. Stems erect, branched, quadrangular, densely puberulent and reddish-brown glandular. Leaves decussate; lamina ovate to broadly ovate, papery, 5–15 × 3–7 cm, apex acuminate, base cuneate to broadly cuneate, margin crenulate, adaxially green, subglabrous to sparsely pubescent, reddish-brown glandular, abaxially light green, subglabrous, densely reddish-brown glandular; lateral veins 3–5-paired; petioles 1–5 cm long. Thyrses terminal and axillary, ca. 10 cm long; cymes 3–7-flowered, peduncles 2–3 mm long, pedicels 4–6 mm long, densely puberulent, glandular puberulent and reddish-brown glandular; bracts broadly ovate, sessile, apex lanceolate, margin entire, 3–10 mm long, bracteoles linear, ca. 1 mm long, ciliate, densely reddish-brown glandular. Calyx campanulate, ca. 2 mm long, densely glandular puberulent and reddish-brown glandular outside; 2-lipped to 1/2 its length, teeth triangular, apex acute, fruiting calyx dilated to ca. 5 mm long. Corolla 5–6 mm long, declinate, pubescent and reddish-brown glandular outside; tube 3–3.5 mm long, white, saccate abaxially near base, ca. 2 mm in diameter, attenuating gradually towards throat to ca. 1 mm in diameter; apex 2-lipped, light bluish-purple, posterior lip 4-lobed, ca. 3.5 × 3.5 mm, reflexed, lobes oblong, apex acute, anterior lip entire, subrounded, concave, straight, 2–3.5 mm in diameter. Stamens 4, included; anther cells 2, confluent, divergent; filaments pubescent at base. Style included, glabrous, apex slightly subequally 2-lobed. Mericarps 4, ochre-yellow, oblong, 1.4–1.55 mm long, ca. 1 mm wide, smooth and glabrous.

##### Phenology.

Flowering from July to September, fruiting from August to October.

##### Distribution and habitat.

Currently, *I.attenuatus* is only known from the Fanjing Mountain in Guizhou Province, China (Fig. 1). The new species usually grows in evergreen broadleaf forests at altitudes of 1600–2000 m.

##### Etymology.

The specific epithet refers to the gradually attenuating corolla tube of the new species towards the throat.

##### Chinese name (assigned here).

jiàn xiá xiāng chá cài (渐狭香茶菜).

##### Additional specimens examined.

China – **Guizhou Prov.** • Jiangkou County, vicini­ty of Jinding along the crest of the Fanjing Shan Mountain Range; alt. 2000–2300 m; 28–29 Aug 1986; *Sino-American Guizhou Botanical Expedition 673* (L3902407!, PE00833681!); • ibid.; alt. 1707 m; 20 Oct 2017; Y.P. Chen & L. Chen EM429 (KUN!).

#### ﻿ Isodon gongshanensis

Taxon classificationPlantaeLamialesLamiaceae

Y.P.Chen & C.L.Xiang sp. nov.

CF5229EA-17B9-59F7-8452-ED44D5A516B5

urn:lsid:ipni.org:names:77348681-1

Fig. 3

##### Type.

China – **Yunnan Prov.** • Gongshan County, Dulongjiang Town; alt. 2915 m; 27°50'33.93″N, 98°27'27.85″E; at the streamside in forests; 15 Aug 2020; *Y.P. Chen et al. EM1570* (holotype: KUN1628216!; isotypes: K!, KUN1628214!, PE!).

##### Diagnosis.

*Isodongongshanensis* is morphologically similar to *I.rosthornii*, but differs by having lanceolate (vs. triangular) and longer (ca. 1 mm vs. ca. 0.5 mm long) teeth of the posterior calyx lip, longer (6–7 mm vs. ca. 5 mm long) corollas with a pink (vs. white) posterior lip, but without spots (vs. with reddish-purple spots) and an ovary without glands (vs. with glands).

Herbs perennial, 50–150 cm tall. Stems erect, branched, quadrangular, 4-sulcate, often claret, subglabrous to densely strigose. Leaves decussate; lamina ovate to broadly ovate, papery, 5–12 × 3–7 cm, apex acuminate, base cuneate to broadly cuneate, margin crenulate, adaxially green, sparsely strigose and glandular, abaxially light green, subglabrous, densely glandular; lateral veins 3–4-paired; petioles 1–5 cm long, claret or green, strigose. Thyrses terminal and axillary, 10–20 cm long; cymes 3–7-flowered, peduncles 1–2 mm long, pedicels 3–5 mm long, strigose; bracts ovate to broadly ovate, 2–30 mm long, apex lanceolate, margin crenulate or entire, petioles 0–5 mm long, bracteoles linear, ca. 1 mm long. Calyx campanulate, ca. 2.5 mm long, strigose and glandular outside; 2-lipped to 1/2 its length, teeth narrowly triangular, apex acuminate, fruiting calyx dilated to ca. 5 mm long, posterior lip strongly reflexed. Corolla white, 6–7 mm long, declinate, strigose and glandular outside; tube 2.5–3 mm long, white, saccate abaxially near base, 1.5–2 mm in diameter; apex 2-lipped, posterior lip pink, 4-lobed, ca. 4 × 4 mm, reflexed, lobes subrounded, anterior lip entire, subrounded, concave, navicular, straight, 3.5–4 mm in diameter. Stamens 4, included; anther cells 2, confluent, divergent; filaments pubescent at base. Style included, glabrous, apex slightly subequally 2-lobed. Ovaries glabrous. Mericarps not seen.

##### Phenology.

Flowering from July to September, fruiting from August to October.

##### Distribution and habitat.

*Isodongongshanensis* is only known from Gongshan County in Yunnan Province, southwest China (Fig. 1). The new species usually grows in mixed needle-leaf and broadleaf forests at altitudes of 2700–2900 m.

##### Etymology.

The specific epithet is derived from the type locality of the new species, i.e. Gongshan County in Yunnan Province, China.

##### Chinese name (assigned here).

gòng shān xiāng chá cài (贡山香茶菜).

##### Additional specimens examined.

China – **Yunnan Prov.** • Gongshan County, Cikai Town, Danzhu Village; alt. 2787 m; 27°37'17.14″N, 98°38'1.04″E; 25 Sep 2022; *Y.J. Zhao et al. 22ZYJ023* (KUN); • Gongshan County, Dulongjiang Town, near the Dulongjiang Tunnel; 2 Jul 2015; *Y.P. Chen & R.L. Stubbs EM203* (KUN!); • ibid.; 16 Oct 2019; *L.Q. Jiang & Y.Y. Li LJ28* (KUN!).

#### ﻿ Isodon sukungii

Taxon classificationPlantaeLamialesLamiaceae

Y.P.Chen & C.L.Xiang sp. nov.

AC63D57F-CB50-5ED8-BFC8-3B6C283D3B0A

urn:lsid:ipni.org:names:77348682-1

Fig. 4

##### Type.

China – **Sichuan Prov.** • Muli County, Sanjiaoya Town, on the way from Biji to Guoquanyan, amongst the thickets on the dry valley slope; 28°04'58.50″N, 101°28'8.77″E; alt. 2041 m; 13 Oct 2018; *Y.P. Chen et al. EM666* (holotype: KUN1628218!; isotypes: K!, KUN1628217!, PE!).

##### Diagnosis.

*Isodonsukungii* is morphologically similar to *I.tenuifolius* but differs by having longer laminae (1–4 cm vs. 0.5–1 cm long) with serrate (vs. entire) margins, cymes that form panicles (vs. single cymes that do not form thyrses or panicles) and a longer corolla (6–7 mm vs. 4 mm long) with a straight (vs. strongly reflexed) anterior lip.

Shrubs 30–120 cm tall. Stems erect, much branched; branches brown, decorticate, subterete, glabrescent; branchlets brown, obtusely 4-angled, densely grey tomentose. Leaves decussate; lamina ovate to oblong, thick papery, 1–4 × 0.5–2 cm, apex acute, base cuneate to subrounded, margin serrate, rarely entire, adaxially green, sparsely minute grey tomentose and glandular, abaxially white, densely grey tomentose and glandular, lateral veins 3–5-paired, conspicuously elevated abaxially; petioles 0.3–1 cm long, densely grey tomentose. Cymes 3–7-flowered, often forming secund panicles up to 20 cm long; bracts leaf-like, gradually reduced towards apex, margin entire, longer than cymes, upper ones sessile, lanceolate, bracteoles linear, ca. 1 mm long; peduncles 1–3 (–5) mm long, pedicels 1–2 mm long, densely grey tomentose. Calyx campanulate, 2.5–3 mm long, densely grey tomentose and glandular outside, slightly 2-lipped to 1/3 its length; teeth subequal, ovate-triangular, apex acute, fruiting calyx slightly dilated to ca. 4 mm long. Corolla white, 6–7 mm long, declinate, pubescent and glandular outside; tube 3.5–4 mm long, saccate abaxially near base, ca. 1.5 mm in diameter; apex 2-lipped, posterior lip 4-lobed, dotted with reddish-purple spots, ca. 2.5 mm long, reflexed, lobes subrounded, anterior lip entire, subrounded, concave, navicular, straight, 2.5–3 mm in diameter. Stamens 4, included; anther cells 2, confluent, divergent; filaments pubescent at base. Style included, glabrous, apex slightly subequally 2-lobed. Mericarps 4, brown, oblong, 1.25–1.35 mm long, 0.85–0.95 mm wide, smooth and glabrous.

##### Phenology.

Flowering from July to November, fruiting from September to December.

##### Distribution and habitat.

*Isodonsukungii* is widely distributed in the dry valley along Min River, a tributary of the Yangtze River, in Sichuan Province, southwest China (Fig. 1). The new species usually grows on open dry slopes with savannah-like vegetation at altitudes of 1600–2700 m.

##### Etymology.

The new species is named after the late Prof. Su-Kung Wu, who is one of the earliest Chinese botanists to explore the plant diversity of Muli County.

##### Chinese name (assigned here).

sù gōng xiāng chá cài (素功香茶菜).

##### Additional specimens examined.

China – **Sichuan Prov.** • Muli County, on the way from Moshuogou to Boao; alt. 1900–2290 m; 6 Sep 1959; *S.K. Wu 2419* (KUN0271016!, KUN0271017!); • Muli County, Boao, Baidiao; alt. 2150 m; 20 Sep 1959; *S.K. Wu 3155* (KUN0271018!, KUN0271019!); • Muli County, Housuo Town, Xiagu Village; 26 Jul 2011; *E.D. Liu et al. 2911* (KUN1278979!); • Muli County, Kala Town, Tianzheng Village; alt. 1966 m; 15 Oct 2018; *Y.P. Chen et al. EM689* (KUN!); • Yanyuan County, Ma’anshan Town, Songlinping Village; alt. 1663 m; 15 Oct 2017; *Y.P. Chen & Z.H. Wang EM419* (KUN!); • Yanyuan County, Jinhe Town; 4 Oct 2020; *L.B. Jia s.n.* (KUN!); • Yajiang County; alt. 2570 m; 18 Aug 2011; *W. Fang et al. FW11269* (KUN1340269!, KUN1340270!); • ibid.; alt. 2711 m; 28 Aug 2020; *Y.P. Chen et al. EM1711* (KUN!).

## Supplementary Material

XML Treatment for Isodon attenuatus

XML Treatment for Isodon gongshanensis

XML Treatment for Isodon sukungii

## ﻿Additional information

### Author ORCIDs

Ya-Ping Chen https://orcid.org/0000-0002-7502-1848

Alan J. Paton https://orcid.org/0000-0002-6052-6675

Chun-Lei Xiang https://orcid.org/0000-0001-8775-6967

### Data availability

All of the data that support the findings of this study are available in the main text.
